# Synthesis and *in Vitro* Antioxidant Activity Evaluation of 3-Carboxycoumarin Derivatives and QSAR Study of Their DPPH• Radical Scavenging Activity

**DOI:** 10.3390/molecules171214882

**Published:** 2012-12-13

**Authors:** Francisco J. Martínez-Martínez, Rodrigo Said Razo-Hernández, Ana Lilia Peraza-Campos, Manuel Villanueva-García, Maria Teresa Sumaya-Martínez, Daniel Jaramillo Cano, Zeferino Gómez-Sandoval

**Affiliations:** 1 Facultad de Ciencias Químicas, Universidad de Colima, kilómetro 9 carretera Colima-Coquimatlán, Col., México, C.P. 28400, Mexico; E-Mails: rrazo@ucol.mx (R.S.R.-H.); peraza@ucol.mx (A.L.P.-C.); jaracano@ucol.mx (D.J.C.); 2 Asociación de Jubilados de la Universidad de Guanajuato, Paseo de la Presa No 77, Guanajuato, Gto., México, C.P. 36000, Mexico; E-Mail: villagm@ugto.mx; 3 Secretaria de Investigación y Posgrado, Universidad Autónoma de Nayarit, Ciudad de la Cultura “Amado Nervo”, Boulevard Tepic-Xalisco S/N, Tepic, Nayarit CP 63190, Mexico; E-Mail teresumaya@hotmail.com

**Keywords:** coumarins, QSAR, DPPH•, artificial neural networks

## Abstract

The *in vitro* antioxidant activities of eight 3-carboxycoumarin derivatives were assayed by the quantitative 1,1-diphenyl-2-picrylhydrazil (DPPH•) radical scavenging activity method. 3-Acetyl-6-hydroxy-2*H*-1-benzopyran-2-one (**C1**) and ethyl 6-hydroxy-2-oxo-2*H*-1-benzopyran-3-carboxylate (**C2**) presented the best radical-scavenging activity. A quantitative structure-activity relationship (QSAR) study was performed and correlated with the experimental DPPH• scavenging data. We used structural, geometrical, topological and quantum-chemical descriptors selected with Genetic Algorithms in order to determine which of these parameters are responsible of the observed DPPH• radical scavenging activity. We constructed a back propagation neural network with the hydrophilic factor (Hy) descriptor to generate an adequate architecture of neurons for the system description. The mathematical model showed a multiple determination coefficient of 0.9196 and a root mean squared error of 0.0851. Our results shows that the presence of hydroxyl groups on the ring structure of 3-carboxy-coumarins are correlated with the observed DPPH• radical scavenging activity effects.

## 1. Introduction

Antioxidants play important roles in preventing diseases induced by reactive oxygen species, which result in oxidative damage, including protein denaturation, mutagenesis and degenerative or pathological events, such as aging, asthma, and cancer. The diversity of structural characteristics in the natural and synthetic coumarins offers a vast field of research for new biological properties of these compounds.

Coumarin derivatives constitute an important class of compounds with varied biological properties such as anti-inflammatory, antibacterial, cytotoxic, anxiolytic, antitumor, anticoagulant, antiemetic and antioxidant activity [1,2,3,4,5,6,7]. Due to thir widespread applications, biological activity evaluation of coumarin derivatives has been a subject of intense investigations.

Here we proposed to measure the antioxidant activity *in vitro* assay of eight 3-carboxycoumarin derivatives with different structural variations for modular replication by the quantitative 1,1-diphenyl-2-picrylhydrazyl (DPPH•) radical scavenging activity method. These is the first time that this measurement has been performed on these compounds, although a similar type of coumarins was reported by Lin *et al.* in 2008 [8].

Quantitative Structure-Activity Relationship (QSAR/QSPR) methodologies are one of the most powerful tools for describing the relationships between biological activity and the physicochemical characteristics of molecules. Current literature demonstrates that almost every area of chemical and life sciences, as well as technology, utilizes quantitative structure-activity/property relationships (QSAR/QSPR) to accelerate product development and increase efficiency. The designs of pharmaceuticals, agrochemicals, and consumer products as well as the assessment of their toxicity and environmental impact have become major areas of application of QSAR/QSPR techniques, whose methods also penetrate into relatively new applications such as materials science and nanotechnology. In terms of methodology development the new trend is the integration of QSAR/QSPR with related computational methods such as virtual screening and molecular dynamics. Such a synergy offers unique opportunities and heralds a new era of computer-aided molecular design [9]. QSAR/QSPR modeling usually consist of four main operations: calculating or measuring a pool of descriptors or other input variables; choosing a small subset of these descriptors that are relevant to the biological activity being modeled (in some cases this step may not be required); generating the often nonlinear relationship between the descriptors and the global material property; and validating the model to assess its reliability, robustness, predictivity, and domain of applicability [10]. Almost all QSPR modeling methods involve some sort of regression. This can be simple least-squares, multiple linear regression (MLR) or, where the structure-property relationship is not linear, a polynomial, bilinear, or neural network method. The simplest QSPR modeling method is known as multiple linear regression, It assumes that the property being modeled is a linear function of the descriptors [11]. To develop a QSAR, a more significant number of compounds is required to develop a meaningful relationship. An often asked question is “how many compounds are required to develop a QSAR?” There is no direct and simple response to this question other than “as many as possible!” To provide some guide, it is widely accepted that between five and ten compounds are required for every descriptor in a QSAR [12]. This does suggest that a one descriptor regression-based QSAR could be developed on five compounds. This is possible, but is very reliant on issues such as data distribution and range. Ideally “many more” compounds are required to obtain statistically robust QSARs, with some modelling techniques being considerably more data hungry than regression analysis. In our case, we have only eight compounds whose biological activities have been determined experimentally in our laboratory. 

Molecular descriptors are formal mathematical representations of a molecule, obtained by a well-specified algorithm, and applied to a defined molecular representation or a well-specified experimental procedure: the molecular descriptor is the final result of a logic and mathematical procedure which transforms chemical information encoded within a symbolic representation of a molecule into a useful number or the result of some standardized experiment. A general consideration about the use of molecular descriptors in modeling problems concerns their information content. This depends on the type of molecular representation used and the defined algorithm for their calculation. There are simple molecular descriptors derived by counting some atom types or structural fragments in the molecule, as well as physicochemical and bulk properties such as, for example, molecular weight, number of hydrogen bond donors/acceptors, number of OH-groups, and so on. Other molecular descriptors are derived from algorithms applied to a topological representation. These are usually termed topological, or 2D-descriptors. Other molecular descriptors are derived from the spatial (x, y, z) coordinates of the molecule, usually called geometrical, or 3D-descriptors; another class of molecular descriptors, called 4D-descriptors, is derived from the interaction energies between the molecule, imbedded into a grid, and some probe. Single indexes derived from a molecular graph are called topological indexes. These are numerical quantifiers of molecular topology that are mathematically derived in a direct and unambiguous manner from the structural graph of a molecule, usually an H-depleted molecular graph. On the other hand many of those descriptors are based directly on the results of quantum-mechanical calculations or can be derived from the electronic wave function or electrostatic field of the molecule [13]. Since the electrophilicity index is a chemical reactivity descriptor and its definition has strong foundation from the density functional theory [14,15], it is appropriate to make use of this descriptor in the QSAR parlance. Recently the electrophilicity index has been used as a possible descriptor of biological activity confirming the fact that the electrophilicity properly quantifies the biological activity. Although there is no one-to-one agreement between AM1 and B3LYP values, the B3LYP method in general provides better estimates of biological activity when compared to the corresponding AM1 values [15]. Within the density functional theory framework some quantum chemical descriptors such the softness, chemical potential and electrophilicity index, where used here because of the good correlation they have shown in the prediction of radical scavenging antioxidant activity [16,17,18,19].

Genetic Algorithms (GA) are powerful computational tools that have been used in many areas of investigation because of their reliable mathematical models. This method is based on the mechanism of evolution of species, the higher descriptor weights (genes) the more preserved in the mathematic model, while the lower weights are eliminated. In this manner, the best mathematical models which represent the observed biological activity (phenotype) are obtained [20,21]. Furthermore, Artificial Neural Networks (ANN) is a computational tool used in the rationale drug design. ANN tries to simulate the human brain mechanism. In this method the basic unit is the neuron and the interconnection of all of them forms the architecture of the neural network. There is a variation of this method called back propagation ANN as well. In this, the output of the network is compared to the real value and then the network weights are adjusted in order to ensure that the error is minimum. This type of neural network is the most frequently used to develop of QSAR and QSPR studies [22,23].

## 2. Experimental Methods and Results

### 2.1. Synthesis and Characterization

**Scheme 1:**
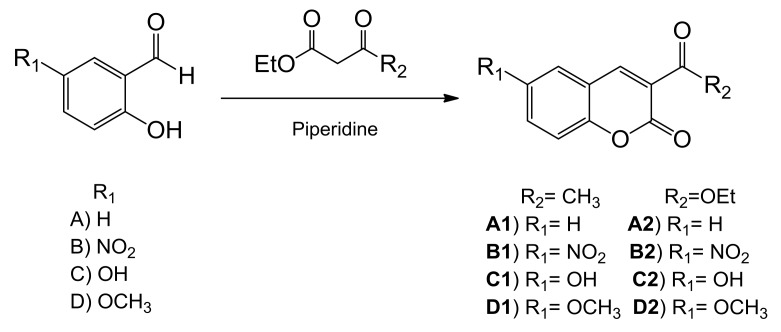
Reaction for the formation of coumarins.

Ethyl esters of 6-R-2*H*-1-benzopyran-2-one-3-carboxylic acids **A1**–**D1** and 3-acetyl-6-R-2*H*-1-benzopyran-2-ones **A2**–**D2** (Scheme 1) were synthesized *via* Knoevenagel condensation. The general reaction between 5-substituted salicylaldehydes **A**–**D** and ethyl acetoacetate or diethyl malonate at refluxing temperature for 24 h gave moderate to good product yields [24,25,26]. The details and spectroscopic data for those compounds are summarized in the Experimental procedures.

### 2.2. DPPH• Radical Scavenging Activity

**Figure 1:**
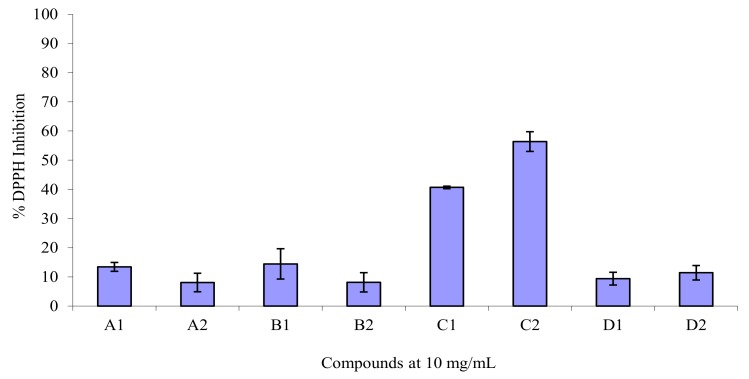
DPPH• radical scavenging activity for 3-carboxycoumarin derivatives at a 10 mg/mL concentration reported as percent of inhibition of DPPH•.

**Figure 2:**
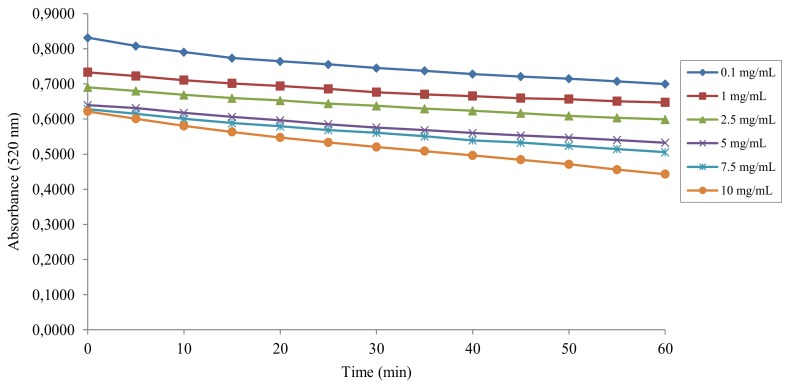
Kinetics of entrapment of DPPH• with respect to the concentration of the compound **C1**.

**Figure 3:**
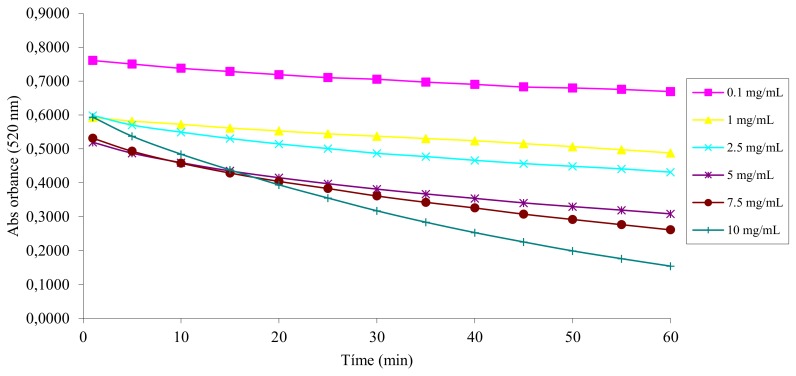
Kinetics of entrapment of DPPH• with respect to the concentration of the compound **C2**.

Antioxidant compounds play an important role as a health-protecting factor. The interaction of the examined compounds with the stable free radical DPPH• was studied. Results of the assays are summarized in Figure 1, Figure 2 and Figure 3. 

Compounds **C1** and **C2** showed the highest radical scavenging activity (Figure 1). For both compounds the interaction was time and concentration dependent (Figure 2 and Figure 3). The time course of DPPH• interaction is affected by various concentrations. In general, this interaction expresses their ability to scavenge free radicals [27,28]. Trials of discoloration of DPPH• at 60 min with different concentrations of compounds **C1** and **C2** in order to verify the dose-effect of the concentration of these compounds on the entrapment of the DPPH• radical [29] are shown below. 

## 3. Computational Details and Results

A conformational study was performed over the eight coumarins (Table 1) using PM3 semi-empirical method as implemented in the SPARTAN′08 code [30,31]. The structures of all conformers of minimum energy were fully optimized without symmetry constrains within the density functional theory methodologies and the resulting ground states were characterized via frequency analysis. In the present work, we have used the hybrid B3LYP [32] functional and the 6-31+G (d,p) basis set [33]. We have included the influence of DMSO solvent using the SMD solvation model [34] implemented in the Gaussian 09 program [35].

Molecular descriptors of all optimized structures were calculated from the DFT context and the DRAGON´05 program [36]. This software includes 20 families of descriptors in the code. Here, we have selected group account, geometrical and molecular property families. These families include a total of 257 descriptors but DRAGON program only gave us 73 descriptors based on the molecular characteristics of our compounds. We calculated the correlation matrix of these 73 descriptors the data analyzer within the Molegro Virtual Docker (MVD) software [37] and obtained nine non-correlated descriptors (see Table 2).

The SPH (spherosity**)** is an anisometry descriptor calculated as a function of the eigenvalues of the covariance matrix calculated from the molecular matrix:

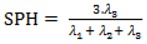
(1)


The spherosity index varies from zero for flat molecules, such as benzene, to one for totally spherical molecules [38]. The Ui (unsaturation index) is a simple information index for unsatured bonds defined as:

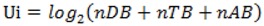
(2)
where *nDB, nTB* and *nAB* are the number of of double, triple and aromatic bonds, respectively [36]. The Hy is the hydrophilic factor descriptor and it’s calculated from Equation (3):

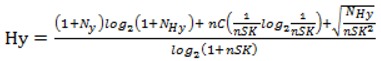
(3)
where *N_Hy_* is the number of hydrophilic groups (-OH, -SH and -NH_2_), *nC* represents the number of carbon atoms and *nSK* stands for all atoms excluding Hydrogen [39]. The AMR (molar refactivity) descriptor is calculated according to the Ghose-Crippen model, based on a group contribution method [40]. The ALOGP descriptor (Ghose-Crippen-Viswanadhan octanol-water partition coefficient) is calculated from the ALOGP model consisting of a regression equation based on the hydrophobicity contribution of 120 atom types [41]. The TPSA (Topological Polar Surface Area) descriptor originally proposed by Ertl P. *et al.* [42] is calculated from Equation (4):

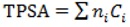
(4)
the *C_i_* term is the contribution of atom *i* to the molecular surface, *n_i_* is the frequency of the atom *i* in the molecule and the sum runs over all types of polar fragments. The TPSA calculation takes into account the contribution of the functional groups containing oxygen and nitrogen atoms to the polarization of the molecular surface as implemented in the DRAGON code [36].

Additionally we calculated quantum chemical descriptors from DFT (Table 2) as total energy (E), dipole moment, hardness (*η*), electrophilicity index (ω), chemical potential (*µ*), softness (*S*) and gap HOMO-LUMO. In this work E, corresponds to the ground state energy of our coumarin molecules and the dipole moment was calculated as implemented in Gaussian 09 [35]. The chemical potential (µ), which is widely used as a descriptor of chemical reactivity, indicates the escape tendency of the electrons and it’s calculated from:

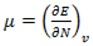
(5)
where *E* is the energy of the system and *N* is the number of electrons [14]. Here we used the finite difference approximation:

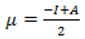
(6)
where *I* is the vertical ionization potential defined as the difference of total energy between cationic structures in the optimized geometry of the neutral compounds and the optimized neutral structures:

*I = E_cat_ − E_neu_* (7)

*A* is the vertical electron affinity defined as the difference of the total energy between the optimized neutral structures and the corresponding anions in the optimized geometry of the neutral compounds:

*A = E_neu_ − E_anion_* (8)

The hardness *(η)* is a global property of the molecular system and measure the resistance imposed by it to any change in its electron distribution:

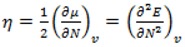
(9)


In the finite difference approximation the above equation is:

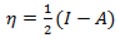
(10)


The softness *(S)* is the inverse of hardness:

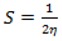
(11)


The electrophilicity index *(ω)* can be determined from chemical potential (*μ*) and hardness (*η*) [14] as:

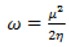
(12)
where *ω* represents the stabilization energy of the molecular system when it is saturated by electrons coming from the surroundings [43].

### 3.1. Genetic Algorithms (GA)

**Figure 4:**
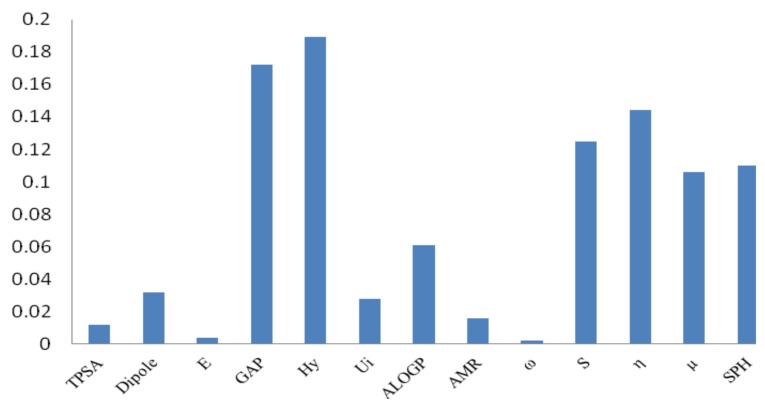
Descriptor weights calculated from *GA* analysis.

We introduced all 13 descriptors into the Neuroshell Predictor program code [44]. According to the GeneHunter Genetic Algorithm [45] implemented in this program we obtained the weights of the molecular descriptors (see Figure 4).

**Figure 5:**
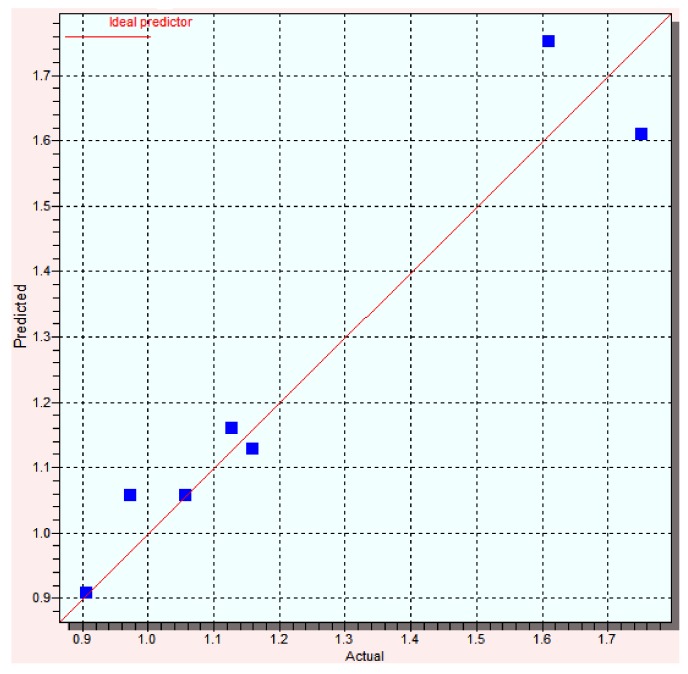
Calculated against experimental antiradical activity.

Figure 5 shows the linear correlation between the log Y_exp_ (actual) and log Y_pred_ calculated by *GA* analysis (predicted). We obtained a coefficient of multiple determinations (R_squared_) of 0.9313, a correlation factor (r) of 0.9658 and a root mean squared error (RMSE) of 0.0786.

R_squared_ is a statistical indicator usually used in multiple regression analysis to compare the reliability of the model with respect to reference points. R_squared_ is defined as:

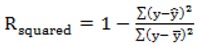
(13)
where *y* is the experimental value, $\hat{y}$ is the value predicted by the model, *y* is the average of all the output values. Furthermore r is a measure of the linear correlation between experimental and predicted values in terms of direction, namely:

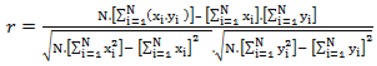
(14)


RMSE is defined as the root mean square of the summation of quadratic terms. These terms correspond to the difference between experimental and predicted data values:

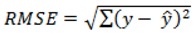
(15)


Experimental and calculated antiradical activity, error and percent error are shown in Table 3. The error is calculated from the difference between experimental (Y_exp_) and calculated (Y_cal_) antiradical activity. Percent error is calculated as:

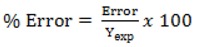
(16)


The highest error value was 8.69% and the lowest one 0%. The average percent error was 3.77%. We propose the construction of a Back Propagation Neural Network (BPNN) with the most important descriptor Hy (Table 4) in order to obtain a mathematical model that fits with the QSAR theory, this is one descriptor per 4 to 10 molecules.

### 3.2. Backpropagation Neural Network

**Figure 6:**
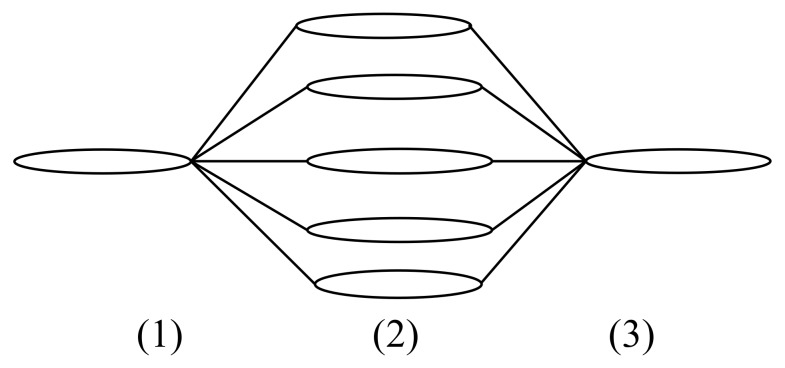
Architecture of the back propagation neural network. Ellipses correspond to neurons and lines represent the interconnection between them. (**1**) Input layer, (**2**) Hidden layer and (**3**) Output layer.

NeuroShell Predictor software [44] was used to build and train our BPNN. The BPNN framework was formed with one input neurons, five hidden neurons and one output neuron (see Figure 6). 

The BPNN model showed that in all the analyzed compounds Hy descriptor is the most important variable in the antiradical activity. The Hy descriptor indicates antiradical activity increases as we incorporate hydrophilic groups to the coumarin molecules. 

**Figure 7:**
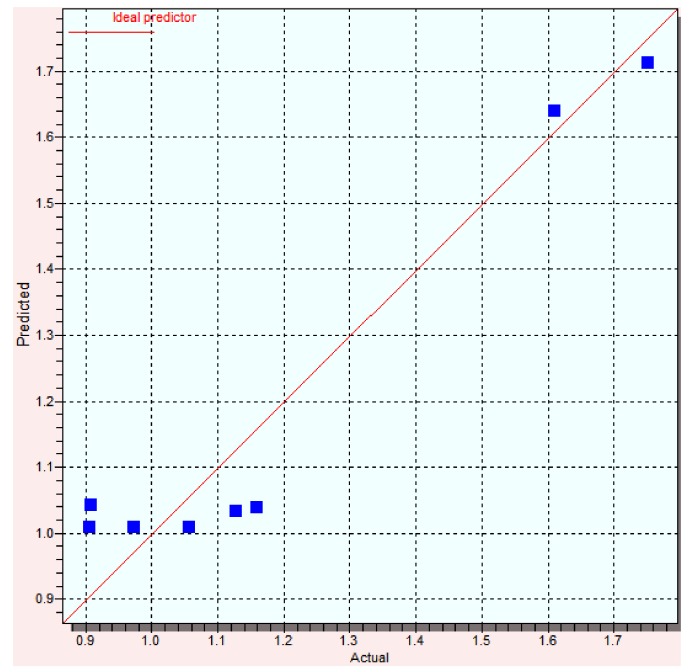
Calculated against experimental antiradical activity.

The linear correlation between log Y_exp_ and log Y_pred_ antiradical activity of coumarins was very successful. The graphic is showed in Figure 7. Here we obtained a R_squered_ of 0.9196, *r* = 0.959 and RMSE = 0.0850.

Experimental and calculated antiradical activity, error and percent error are shown in Table 5. The highest % error value was 14.29% and the lowest one 1.26%. In our opinion the high errors should decrease as the number of molecules is increased. In the BPNN methodology the average percent error was 7.18% which corresponds to a 3.41% higher than the calculated from *GA*.

Determination of reliability of our QSAR model was done by calculating the statistical parameters ${\hat{r}}_{m}^{2}$ and ∆$r_{m}^{2}$ proposed by Roy *et al.* [46,47]. The ${\hat{r}}_{m}^{2}$ value for this mathematical model was 0.8687 and the ∆$r_{m}^{2}$ = 0.0759. For an acceptable QSAR model the average r_m_^2^ must be >0.5 and ∆r_m_^2^ < 0.2, in this terms the QSAR model proposed here was good. In contrast ${\hat{r}}_{m}^{2}$ and ∆r_m_^2^ values for our *GA* model was of 0.9014 and 0.056 respectively, but we have to consider that in *GA* analysis 13 descriptors were used and in the ANN only one. These results show the importance to include ANN with the *GA* methodology. A previous QSAR study [48] made with Multiple Linear Regression and 15 more complex coumarins derivatives they found that the HOMO, LUMO and partial charges in the OH, N and S where the most important descriptors for the development of the antiradical scavenging activity. There’s results concord with ours in the way that Hy take account the functional groups OH, NH_2_ and SH. Also in our study we validated our model with the statistical parameters ${\hat{r}}_{m}^{2}$ and ∆$r_{m}^{2}$ [44,45] that are a rigorous method for QSAR evaluation. 

It’s important to mention that the **C1** and **C2** compounds show the highest antiradical activities because both possess an -OH hydrophilic group. This functional group increases the Hy value in such a way that we could say that -OH group is crucial for antiradical activity of coumarins. 

## 4. Experimental

### 4.1. General

All chemicals and solvents were of reagent grade and used as received. Melting points were measured on an Electrothermal IA 9100 apparatus and were uncorrected. IR spectra were recorded neat using a Varian 3100 FT-IR with ATR system Excalibur Series spectrophotometer. Mass spectra were obtained in a Bruker Esquire 6000 spectrometer with an electron ionization mode. ^1^H and ^13^C-NMR spectra were recorded on a Varian Mercury 300 (^1^H, 300.08; ^13^C, 75.46 MHz) instrument in CDCl_3_ solutions or DMSO-d_6_, measured with SiMe_4_ as the internal reference, chemical shifts are in ppm and ^n^*J*(H-H) in Hertz.

#### General Procedure for the Synthesis of Coumarin Derivatives

The starting coumarins **1A**–**2D** were synthesized by Knovenagel cyclization (Scheme 1) between substituted salicylaldehydes (4 mmol) and ethyl acetoacetate (4 mmol) or diethyl malonate (4 mmol) with catalytic amounts of piperidine in ethanol (20 mL), according to the methodology reported elsewhere [24,25,26]. 

*3-Acetyl-2H-1-benzopyran-2-one* (**A1**): Yellow solid. Yield 67.3%. m.p: 118–122 °C, IR (neat), ν (cm^−1^): 1719 (OC=O), 1680 (C=O), 1196 and 1161 (C-O). ^1^H-NMR (δ ppm, CDCl_3_): 8.63 (s, 1H, H-4), 7.92 (d, 1H, H-5, ^3^*J* = 7.7 Hz), 7.72 (dd, 1H, H-7, ^3^*J* = 7.6, 7.3 Hz), 7.43 (dd, 1H, H-8, ^3^*J* = 7.3 Hz), 7.39 (dd, 1H, H-6, ^3^*J* = 7.7, 7.6 Hz), 2.56 (s, 3H, H-12). ^13^C-NMR (δ ppm, CDCl_3_): 195.1 (C-11), 158.5 (C-2), 154.6 (C-9), 147.1 (C-4), 134.5 (C-7), 130.8 (C-5), 124.9 (C-6), 124.4 (C-3), 118.2 (C-10), 116.1 (C-8), 30.1 (C-12). C_11_H_8_O_3_ 188.05. *m/z* = 188.1 (M, 50%), 173.2 (100%), 145.3 (11.9%), 118.3 (11.1%).

*3-Acetyl-6-nitro-2H-1-benzopyran-2-one* (**B1**): Yellow solid. Yield 54.6%. m.p: 200–203 °C; IR (neat), ν (cm^−1^): 1745 (OC=O), 1676 (C=O), 1530 and 1341 (C-NO_2_), 1275 y 1209 (C-O). ^1^H-NMR (δ ppm, CDCl_3_): 8.58 (d, 1H, H-5, ^4^*J* = 2.6 Hz); 8.55 (s, 1H, H-4); 8.50 (dd, 1H, H-7, ^3^*J* = 9.1, ^4^*J* = 2.6 Hz), 7.52 (d, 1H, H-8, ^3^*J* = 9.1 Hz), 2.73 (s, 3H, H-12). ^13^C-NMR (δ ppm, CDCl_3_): 194.5 (C-11), 158.6 (C-2), 157.9 (C-9), 146.1 (C-4), 144.6 (C-6), 128.8 (C-7), 126.5 (C-3), 126.1 (C-5), 118.4 (C-10), 118.2 (C-8), 30.7 (C-12). C_11_H_7_NO_5_ 233.03. *m/z* = 233.0 (M, 43.9%), 218.3 (100%), 172.3 (19.3%), 144.2 (5.0%).

*3-Acetyl-6-hydroxy-2H-1-benzopyran-2-one* **(C1**): Yellow solid. Yield 90%. m.p: 247–248 °C; IR (neat), ν (cm^−1^): 3155 (O-H), 1736 (OC=O), 1643 (C=O). ^1^H-NMR(δ ppm, DMSO_d6_): 9.89 (s, 1H, OH), 8.53 (s, 1H, H-4), 7.20 (d, 1H, H-5, ^4^*J* = 2.9 Hz); 7.14 (dd, 1H, H-7, ^3^*J* = 8.8, ^4^*J* = 2.9 Hz), 7.29 (d, 1H, H-8, ^3^*J* = 8.8 Hz), 2.54 (s, 3H, H-12). ^13^C-NMR (δ ppm, DMSO-d_6_): 196.0 (C-11), 159.9 (C-2), 154.6 (C-6), 148.7 (C-9), 147.7 (C-4), 125.1 (C-3), 123.4 (C-7), 119.3 (C-10), 117.2 (C-5), 114.8 (C-8), 30.7 (C-12). C_11_H_8_O_4_ 204.04. *m/z* = 204.1 (M, 86.0%), 189.1 (100%), 161.2 (19.0%), 134.2 (33.4%).

*3-Acetyl-6-methoxy-2H-1-benzopyran-2-one* (**D1**): Yellow solid. Yield 89%. mp 180–183 °C. IR ν (cm^−1^): 1723 (OC=O), 1677 (C=O), 1226, 1197 (C-O).^1^H-NMR (δ ppm, CDCl_3_): 8.44 (s, 1H, H4), 7.28 (d, H-8, 1H, ^3^*J* = 9.1,), 7.20 (dd, 1H, H-7, ^3^*J* = 9.1, ^4^*J* = 2.9 Hz), 7.02 (d, 1H, H-5, ^4^*J* = 2.6 Hz), 3.85 (s, 3H, OCH_3_), 2.70 (s, 3H, CH_3_); ^13^C-NMR (δ ppm, CDCl_3_): 195.9 (C11), 159.7 (C2), 156.6 (C6), 150.1 (C10), 147.6 (C4), 124.8 (C3), 123.2 (C7), 117.9 (C5), 118.7 (C9), 111.3 (C8), 56.1 (OCH_3_), 30.9 (CH_3_). C_12_H_10_O_4_ 218.06 *m/z* = 218.0 (M, 60.0%), 203.0 (100%), 175.0 (16.6%), 148.0 (15.3%). EA (%) calculated for C_12_H_10_O_4_: 66.05 C, 4.62 H; found: 66.04 C, 4.61 H.

*Ethyl 2H-1-benzopyran-2-one-3-carboxyate* (**A2**): White solid. Yield 90%. m.p. 91–92 °C, IR (neat), ν (cm^−1^): 1605 (OC=O), 11758 (C=O), 1196 y 1161 (C-O). ^1^H-NMR (δ ppm, DMSO-d_6_): 8.49 (s, 1H, H-4), 7.93 (dd, 1H, H-5), 7.39 (dt, 1H, H-6), 7.74 (dt, 1H, H-7), 7.45 (dd, 1H, H-8), 4.30 (q, 2H, O-CH_2_), 1.39 (t, 3H, -CH_3_). ^13^C-NMR (δ ppm, DMSO-d_6_): 163.0 (C-11), 156.4 (C-2), 154.5 (C-9), 149.0 (C-4), 134.7 (C-7), 130.7 (C-5), 125.3 (C-6), 118.2 (C-3), 118.3 (C-10), 116.6 (C-8), 61.7 (O-CH_2_), 15.5 (-CH_3_). C_12_H_10_O_4_ 218.06. *m/z* = 219.1 (M, 100%), 173.2 (76%), 146.2 (87.9%), 118.2 (27.4%).

*Ethyl 6-nitro-2H-1-benzopyran-2-one-3-carboxyate* **(B2**): Yellow solid. Yield 89%. m.p = 191–192 °C, *IR* (neat), ν (cm^−1^): O-C=O (1716), C=O (1746). ^1^H-NMR(δ ppm, CDCl_3_): 8.90 (d, 1H, H-5, ^4^*J* = 3.0 Hz); 8.93 (s, 1H, H-4); 8.50 (dd, 1H, H-7, ^3^*J* = 9.0, ^4^*J* = 3.0 Hz), 7.65 (d, 1H, H-8, ^3^*J* = 9.0 Hz), 4.32 (q, 2H, O-CH_2_), 1.33 (t, 3H, -CH_3_). ^13^C-NMR (δ ppm, CDCl_3_): 162.5 (C-11), 158.5 (C-2), 155.2 (C-9), 147.1 (C-4), 148.8 (C-6), 128.8 (C-7), 118.0 (C-3), 125.4 (C-5), 120.7 (C-10), 118.3 (C-8), 62.7 (O-CH_2_), 14.3 (-CH_3_). C_12_H_9_NO_6_ 263.04. *m/z* = 262.9 (M, 33.0%), 218.2 (91.9%), 191.0 (100%), 161.2 (98.0%).

*Ethyl 6-hydroxy-2H-1-benzopyran-2-one-3-carboxyate* (**C2**): Beige solid, 91%, m.p. = 205–206 °C, *IR* (neat,) ν (cm^−1^): (O-H) 3324, (O-C=O) 1707, (C=O) 1722. ^1^H-NMR (δ ppm, DMSO-d_6_): 9.93 (s, 1H, OH), 8.66 (s, 1H, H4), 7.30 (d, 1H, H-8, ^3^*J* = 8.8), 7.21 (d, 1H, H-5, ^4^*J* = 2.9), 7.17 (dd, 1H, H-7, ^3^*J* = 8.8, ^4^*J* = 2.9 Hz), 4.29 (q, 2H, OCH_2_-, ^3^*J* = 7.1), 1.31 (t, 3H, -CH_3_, ^3^*J* = 7.1), ^13^C-RMN (δ ppm, DMSO-d_6_): 167.4 (C-11), 159.4 (C-2), 154.5 (C-9), 147.2 (C-4), 140.9 (C-6), 117.1 (C-7), 119.4 (C-3), 113.3 (C-5), 119.7 (C-10), 120.2 (C-8), 44.1 (O-CH_2_), 14.2 (-CH_3_). C_12_H_10_O_5_ 234.05. *m/z* = 234.0 (M, 67.7%), 189.1 (36.1%), 161.8 (100.0%), 134.2 (31.3%)

*Ethyl 6-methoxy-2H-1-benzopyran-2-one-3-carboxyate* (**D2**): Yellow solid, 92%, m.p. = 142–143 °C, *IR* (neat), ν (cm^−1^): (O-C=O)1733, (C=O)1740. ^1^H-NMR (δ, ppm, CDCl_3_): 8.45 (s, 1H, H-4), 7.26 (d, H-8, 1H, ^3^*J* = 5.0), 7.20 (dd, 1H, H-7, ^3^*J* = 5.0, ^4^*J* = 2.9, Hz), 6.99 (d, 1H, H-5, ^4^*J* = 2.9 Hz), 3.89 (s, 3H, OCH_3_) 4.38 (q, H, OCH_2_, ^3^*J* = 7.0, Hz), 1.47 (s, 3H, -CH_3_, ^3^*J* = 7.0 Hz); ^13^C-NMR (δ ppm, CDCl_3_): 163.3 (C-11), 157.1 (C-2), 156.4 (C-9), 148.6 (C-4), 149.9 (C-6), 118.0 (C-7), 118.3 (C-3), 110.8 (C-5), 118.6 (C-10), 122.8 (C-8), 62.1 (O-CH_2_), 56.1 (CH_3_O-), 14.4 (-CH_3_). C_13_H_12_O_5_ 248.07. *m/z* = 248.2 (M, 93.1%), 203.1 (32.6%), 176.2 (100%), 148.2 (23.7%).

### 4.2. Antiradical Activity Measurement with the DPPH• Assay

The antiradical activity of compounds **A1**–**D2** was estimated according to a slight modification of the procedure reported by Morales and Jimenez-Perez [27]. Dilutions in DMSO solvent at 10 mg/mL of the eight compounds were prepared. An aliquot of each sample (50 μL) was added to a solution of 1,1-diphenyl-2-picrylhydrazyl (DPPH•) radical (250 µL) prepared fresh daily, at a concentration of 74 mg/L in ethanol. The mixtures (200 µL) were placed in a 96-well microplate and absorbance at time zero was immediately measured using a UV wavelength of 520 nm. Measurement were performed every 5 min for 60 min. Antiradical activity evaluation for compounds was measured in terms of absorbance decrease at 520 nm of the DPPH• ethanolic solution produced by the effect of each compound as a result of their ability to donate a hydrogen giving place to the reduced form of DPPH•. 6-hydroxy-2,5,7,8-tetramethylchroman-2-carboxylic acid (Trolox) was used as standard molecule. The antiradical activity for each compound was determinate in Trolox equivalent antioxidant capacity (TEAC). The DPPH• solution in presence of DMSO and in the absence of coumarins was tested and used as a negative control. A null DPPH• free radical scavenging for the DMSO was verified. In all experiments, samples were analyzed in triplicate, and mean values ± SD were recorded in order to present the activity for each compound and be able to evaluate the structure-activity relationships.

## 5. Conclusions

In *GA* analysis we obtained an average percent error of 3.77% while in BPNN the average percent error was 7.18%. This result indicate that the combination of the two methodologies optimize the creation of QSAR models. The *GA* allows finding the most important descriptor for the development of the antiradical activity and ANN improves our model with the use of only one molecular descriptor to obtain accurate prediction values. The presence of hydroxyl groups on the ring structure of 3-carboxycoumarins is correlated with their DPPH• radical scavenging effects. The mixed QSAR model showed that Hy could indicate that antiradical activity would increase as we incorporate hydroxyl groups in the coumarin molecules. According to ${\hat{r}}_{m}^{2}$ and ∆$r_{m}^{2}$ obtained for ANN the mathematical model proposed in this work has good predictive ability.

## Figures and Tables

**Table 1:** Coumarin compounds and their experimental antiradical activity expressed as equivalents in % DPPH• inhibition.

Compound	R_2_	R_1_	% DPPH• inhibition	Log Y_exp_
A1	COOCH_3_	H	13.42	1.13
B1	COOCH_3_	NO_2_	14.43	1.16
C1	COOCH_3_	OH	40.67	1.61
D1	COOCH_3_	OCH_3_	9.39	0.97
A2	COOEt	H	8.06	0.91
B2	COOEt	NO_2_	8.10	0.91
C2	COOEt	OH	56.39	0.75
D2	COOEt	OCH_3_	11.41	1.06

**Table 2:** Descriptors used in our study.

PROGRAM	DESCRIPTOR	TYPE	DESCRIPTION
DRAGON	SPH	Geometrical	Spherosity
DRAGON	Ui	Molecular Properties	Unsaturation index
DRAGON	Hy	Molecular Properties	Hydrophilic factor
DRAGON	AMR	Molecular Properties	Molar refractivity
DRAGON	ALOGP	Molecular Properties	Ghose-Crippen-Viswanadhan octanol-water partition coefficient
DRAGON	TPSA	Molecular Properties	Topological Polar Surface Area
GAUSSIAN	E	Quantum-Chemical	Total Energy
GAUSSIAN	Dipole	Quantum-Chemical	Dipole Moment
GAUSSIAN	η	Quantum-Chemical	Hardness
GAUSSIAN	ω	Quantum-Chemical	Electrophilicity
GAUSSIAN	µ	Quantum-Chemical	Chemical potential
GAUSSIAN	S	Quantum-Chemical	Softness
GAUSSIAN	Gap HOMO-LUMO	Quantum-Chemical	Energy difference LUMO-HOMO

**Table 3:** log Y_exp_ and log Y_pred_ antiradical activity, error and % error values of coumarin compounds.

Compound	Log Y_exp_	Log Y_pred_	Error	% Error
A1	1.13	1.16	−0.03	2.65
B1	1.16	1.13	0.03	2.58
C1	1.61	1.75	−0.14	8.69
D1	0.97	1.05	−0.08	8.24
A2	0.91	0.91	0.00	0.00
B2	0.91	0.91	0.00	0.00
C2	1.75	1.61	0.14	8.00
D2	1.06	1.06	0.00	0.00

**Table 4:** Hydrophilic Factor (Hy).

Compound	Hy
**A1**	−0.766
**B1**	−0.634
**C1**	−0.200
**D1**	−0.734
**A2**	−0.734
**B2**	−0.621
**C2**	−0.198
**D2**	−0.709

**Table 5:** Experimental and calculated antiradical activity, error and % error values of coumarin compounds with BPNN model.

Compound	Log Y_exp_	Log Y_pred_	Error	% Error
1	1.13	1.03	0.1	8.85
B1	1.16	1.04	0.12	10.34
C1	1.61	1.64	−0.03	1.86
D1	0.97	1.01	−0.04	4.12
A2	0.91	1.01	−0.1	10.99
B2	0.91	1.04	−0.13	14.29
C2	1.75	1.71	0.04	2.29
D2	1.06	1.01	0.05	4.72
